# Phylogenetic relationship of *Ornithobacterium rhinotracheale* isolated from poultry and diverse avian hosts based on 16S rRNA and *rpoB* gene analyses

**DOI:** 10.1186/s12866-019-1395-9

**Published:** 2019-02-06

**Authors:** Inês M. B. Veiga, Dörte Lüschow, Stefanie Gutzer, Hafez M. Hafez, Kristin Mühldorfer

**Affiliations:** 1 Present Address: Institute of Animal Pathology, Bern, Switzerland; 2 Institute of Poultry Diseases, Freie Universität Berlin, Berlin, Germany; 3 Department of Wildlife Diseases, Leibniz Institute for Zoo and Wildlife Research, Berlin, Germany

**Keywords:** Bacteria, Flavobacteriaceae, *Ornithobacterium*, ORT, Phylogeny, Birds, Diagnostics, PCR

## Abstract

**Background:**

*Ornithobacterium* (*O.*) *rhinotracheale* is an emerging bacterial pathogen in poultry and not fully understood to date. Because of its importance particularly for the global turkey meat industry, reliable diagnostic and characterization methods are needed for early treatment and in future for better vaccine production. The host range of birds infected by *O. rhinotracheale* or carrying the bacterium in their respiratory tract has constantly increased raising important epidemiological and taxonomic questions for a better understanding of its diversity, ecology and transmission cycles. The purpose of this study was to introduce partial *rpoB* gene sequencing for *O. rhinotracheale* into routine diagnostics to differentiate strains isolated from poultry and more diverse avian hosts (i.e., birds of prey, corvids and pigeons) and to compare phylogenetic relationships with results from 16S rRNA gene analysis and multilocus sequence typing (MLST).

**Results:**

Partial 16S rRNA gene analysis revealed a high level of homogeneity among the 65 investigated *O. rhinotracheale* sequences with similarity values ranging from 98.6 to 100% between sequences from non-galliform and poultry species. The corresponding *rpoB* gene sequences were heterogeneous and ranged in their similarity values from 85.1 to 100%. The structure of the *rpoB* tree was in strong correlation with previous MLST results revealing three main clusters A (poultry and birds of prey), B (poultry, birds of prey and corvids) and C (pigeons), which were clearly separated from each other.

**Conclusions:**

By using partial sequences from a single gene, the *rpoB* gene analysis is in good agreement with MLST results with a slight decrease in resolution to distinguish more similar strains. The present results provide strong evidence that traditional phenotypic and genetic methods may not properly represent the heterogeneous group of bacteria classified as *O. rhinotracheale*. From housekeeping gene analyses, it is very likely that the genus *Ornithobacterium* includes additional species and partial *rpoB* gene sequencing can be recommended as fast, cost-effective and readily available method to identify strains and differentiate between *O. rhinotracheale* and *Ornithobacterium*-like bacteria.

## Background

*Ornithobacterium* (*O.*) *rhinotracheale* is a relatively novel, emerging bacterial pathogen in turkeys and chickens that causes high economic losses to the commercial poultry production. It was first recognized and taxonomically classified in the early 1990’s [1, 2] and has been isolated from poultry flocks worldwide [3, 4].

The genus *Ornithobacterium* belongs to the family of the Flavobacteriaceae [5], which - besides others - also includes the genus *Riemerella* with *R. anatipestifer* [6, 7] and the genus *Coenonia* with *C. anatina* [8]. Both bacterial species are also important poultry pathogens mainly of domestic ducks and geese. Besides a new species proposed as *Candidatus Ornithobacterium hominis* sp. nov. [9], *O. rhinotracheale* is the only bacterial species described within the genus *Ornithobacterium* but not fully understood to date. Because of its veterinary importance particularly for the global turkey meat industry [10] the need for reliable diagnostic and characterization methods is obvious for early treatment. Its cultural characteristics and fastidious requirements (i.e., small colony size, slow growth, enriched media and capnophilic incubation), however, may challenge bacterial isolations and reduce the detection rates [11, 12]. Therefore, molecular detection of *O. rhinotracheale* DNA from tissues or swabs targeting the 16S rRNA gene with specific primers is frequently used in routine diagnostics, but 16S sequences often lack the resolution to capture heterogeneity among the strains [13–15].

Over the past few decades, the host range of birds infected by *O. rhinotracheale* or carrying the bacterium in their respiratory tract has constantly increased [16]. The presence of *O. rhinotracheale* in apparently healthy, captive and free-ranging non-galliform species raises important epidemiological and taxonomic questions for a better understanding of its diversity, ecology and transmission cycles. Multilocus sequence typing (MLST) recently established by Thieme et al. [3, 16] not only revealed specific phylogenetic relationships in non-galliform birds such as pigeons and birds of prey, but also identified few strains from turkeys and chickens that clearly differed from the main poultry cluster. The aim of the present study was to introduce partial *rpoB* gene sequencing for *O. rhinotracheale* into routine diagnostics to differentiate strains isolated from poultry and more diverse avian hosts and to compare the results with those from 16S rRNA gene and MLST analyses. The *rpoB* gene has been proved very useful and powerful for bacterial identification and phylogenetic studies [17–19].

## Methods

### Bacterial strains

Sixty-five strains previously identified as *O. rhinotracheale* were used for genetic analyses (Table 1), including 51 strains that were already investigated by MLST and represent the 31 sequence types (ST) as described by Thieme et al. [3, 16], and six additional strains isolated from turkeys in Portugal (present study). Eight strains were identified only genetically from swab samples of different birds of prey and corvid species [20]. They were included in the *rpoB* gene analysis to investigate mixed bacterial DNA samples, when *O. rhinotracheale* cannot be obtained from bacterial cultures because of its fastidious growth requirements and concomitant fast-growing bacteria.

**Table 1:** *Ornithobacterium rhinotracheale* strains used for 16S rRNA and *rpob* gene analyses

Strain ID	Host	Bird family	MLST	Reference
RefA, RefE, GB 1707/12/2, GB 1707/12/3	Chicken	Phasianidae	ST1	[3], present study
RefB, RefI, RefM, RefP, GB 1312/05/2	Turkey	Phasianidae	ST1	[3], present study
RefC	Chicken	Phasianidae	ST2	[3], present study
RefD, RefH	Turkey	Phasianidae	ST3	[3], present study
RefQ	Chicken	Phasianidae	ST3	[3], present study
GK 1112/96	Pheasant	Phasianidae	ST3	[3], present study
RefF	Turkey	Phasianidae	ST4	[3], present study
RefG	Chicken	Phasianidae	ST5	[3], present study
RefJ, RefK	Chicken	Phasianidae	ST6	[3], present study
RefL	Turkey	Phasianidae	ST6	[3], present study
RefN	Guinea fowl	Numididae	ST7	[3], present study
RefO	Rook	Corvidae	ST8	[3], present study
GB 1312/05/22, GB 371/09/5, GB 804/13/1	Turkey	Phasianidae	ST9	[3], present study
GB 137/10/2	Chicken	Phasianidae	ST10	[3], present study
GB 738/10/1, GB 738/10/3	Turkey	Phasianidae	ST11	[3], present study
GB 1573/11/17	Turkey	Phasianidae	ST12	[3], present study
GB 2399/13	Chicken	Phasianidae	ST13	[3], present study
GB 978/14/1	Turkey	Phasianidae	ST14	[3], present study
GV1	Turkey vulture	Cathartidae	ST15	[3, 20], present study
GV6	Harris’s hawk	Accipitridae	ST16	[3, 20], present study
GV13	Red kite	Accipitridae	ST16	[3, 20], present study
GV9	Common kestrel	Falconidae	ST17	[3, 20], present study
GV10	Peregrine falcon	Falconidae	ST18	[3, 20], present study
GV11	Saker falcon	Falconidae	ST18	[3, 20], present study
GV12	Saker-gyrfalcon	Falconidae	ST18	[3, 20], present study
GV143	Common kestrel	Falconidae	ST19	[3, 20], present study
GV149	Common kestrel	Falconidae	ST20	[3, 20], present study
T85	Pigeon	Columbidae	ST21	[16], present study
T49	Pigeon	Columbidae	ST22	[16], present study
T97	Pigeon	Columbidae	ST23	[16], present study
T91	Pigeon	Columbidae	ST24	[16], present study
T37	Pigeon	Columbidae	ST25	[16], present study
T66, T143	Pigeon	Columbidae	ST26	[16], present study
T52	Pigeon	Columbidae	ST27	[16], present study
T92	Pigeon	Columbidae	ST28	[16], present study
T102	Pigeon	Columbidae	ST29	[16], present study
T203	Pigeon	Columbidae	ST30	[16], present study
165–2/2015	Common buzzard	Accipitridae	ST31	[16], present study
GV22^a^	Northern goshawk	Accipitridae	n.d.	[20], present study
GV37^a^	White-tailed eagle	Accipitridae	n.d.	[20], present study
GV38^a^	Osprey	Accipitridae	n.d.	[20], present study
GV55^a^	Common kestrel	Falconidae	n.d.	[20], present study
R68^a^	Carrion crow	Corvidae	n.d.	[20], present study
R70^a^	Eurasian magpie	Corvidae	n.d.	[20], present study
GV82^a^	Common buzzard	Accipitridae	n.d.	[20], present study
GV89^a^	Sparrow hawk	Accipitridae	n.d.	[20], present study
PTCV-ORT-Mist, PTCV731, PTCV1320, PTCV1556, PTCV1714, PTCV2283	Turkey	Phasianidae	n.d.	present study

*MLST* Multilocus sequence typing, *ST* Sequence type, *n.d.* Not determined

^a^swab sample

### 16S rRNA and *rpoB* gene analyses

Amplification of the *O. rhinotracheale* specific 16S rRNA gene fragment (784 bp) was performed according to Numee et al. [14] with primer sequences described by van Empel and Hafez [11]. The *rpoB* gene fragment (538 bp) was amplified using 1 μM of primers rpoBFla-f (5‘-TCAATTCGTTCTTTGGAAC- 3′) and rpoBFla-r (5‘-GCATCATGTTAGATCCCAT-3′) with cycling conditions as follows: 3 min denaturation at 94 °C, followed by 30 cycles at 94 °C for 30 s, 54 °C for 30 s and 72 °C for 45 s, and a final extension step at 72 °C for 5 min. *rpoB* primers were designed based on published genomic sequences within the family Flavobacteriaceae (including *Riemerella* strains NCTC 11014^T^ and LMG 11607^T^, and *O. rhinotracheale* strain DSM 15997^T^) as described by Christensen and Bisgaard [19]. 16S rRNA and *rpoB* PCR products were gel purified (MinElute Gel Extraction Kit, Qiagen, Hilden, Germany) and Sanger sequenced in both directions at LGC Genomics, Berlin, Germany, using PeakTrace™ Basecaller and the PHRED 20 quality score. The identity of bacterial species was confirmed using BLAST search against the GenBank database.

Phylogenetic analyses of partial 16S rRNA (632 bp) and r*poB* (538 bp) gene sequences were performed with MEGA6 [21] by using the Maximum Likelihood method based on the Jukes-Cantor model [22]. As several *O. rhinotracheale* strains were identical in their partial 16S rRNA and *rpoB* gene sequences, phylogenetic trees were built for 47 out of 65 strains and *Riemerella anatipestifer* DSM 15868^T^ (accession number NC_017045) was included as outgroup. The *O. rhinotracheale* type strain DSM 15997^T^ (accession number NC_018016) was included for sequence similarity comparisons (Table 2).

**Table 2:** *rpoB* gene sequence similarity values of phylogenetic clusters in comparison and with type strain *Ornithobacterium rhinotracheale* DSM 15997^T^

*rpoB* gene sequences	Cluster A	Cluster B	Cluster C	Strain GV37	DSM 15997^T^
Cluster A	94.2 to 100%	87.4 to 89.0%	85.3 to 87.0%	86.2 to 87.4%	87.5 to 87.9%
Cluster B	87.4 to 89.0%	98.5 to 100%	88.5 to 90.0%	86.8 to 87.5%	98.7 to 100%
Cluster C	85.3 to 87.0%	88.5 to 90.0%	98.0 to 100%	85.1 to 85.5%	88.9 to 89.2%
Strain GV37	86.2 to 87.4%	86.8 to 87.5%	85.1 to 85.5%	100%	87.0%

## Results and discussion

**Fig. 1:**
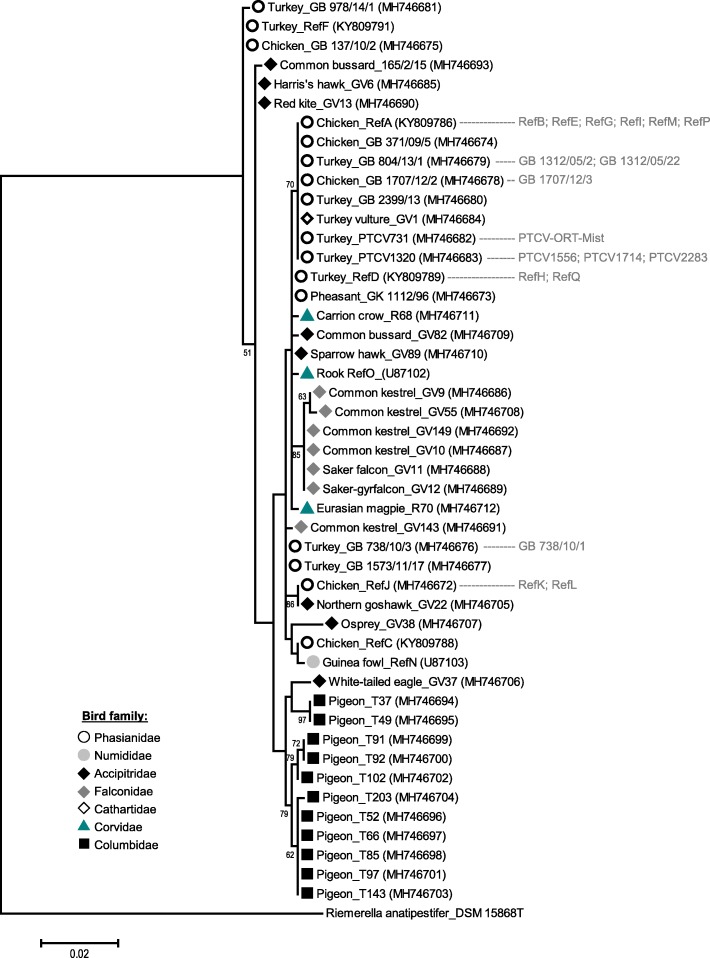
Phylogenetic tree based on partial 16S rRNA gene sequences (632 bp) and constructed in MEGA6 [21] by using the Maximum Likelihood method based on the Jukes-Cantor model [22]. The tree was built with 47 out of 65 *O. rhinotracheale* sequences (remaining identical sequences are indicated in grey) and *Riemerella anatipestifer* DSM 15868^T^ (NC_017045) as outgroup. GenBank accession numbers are given in brackets. The percentage of replicate trees (> 50%) in which the associated taxa clustered together in the bootstrap analysis (100 replicates) is shown next to the branches. Initial tree(s) for the heuristic search were obtained by applying the Neighbor-Joining method to a matrix of pairwise distances estimated using the Maximum Composite Likelihood (MCL) approach. The tree is drawn to scale, with branch lengths measured in the number of substitutions per site

Within the 16S rRNA gene analysis, the 65 *O. rhinotracheale* specific partial sequences showed a high level of homogeneity with similarity values ranging from 98.6 and 98.7% (ten strains from pigeons, GV37 and GV38) to 100% (strains from turkey or chicken and GV1) between non-galliform and poultry species. The structure of the phylogenetic tree (Fig. 1), however, was largely comparable to MLST results [16]. The majority of *O. rhinotracheale* strains were arranged in one cluster and separated from two subsets of three strains each isolated from poultry (family Phasianidae) or birds of prey (family Accipitridae), respectively. One of the *O. rhinotracheale* serotype reference strains (RefF) belonged to the aforementioned subset of the three strains from poultry (including GB 978/14/1 and GB 137/10/2) and formed a separate cluster in previous studies too [14, 15].

The main 16S cluster was split into two subclusters with 47 strains collected from poultry, birds of prey and corvids, which are more closely related to each other and comprise a high diversity of bird families and species. In contrast, the second subcluster only involved 11 strains from pigeons and one strain from a white-tailed eagle (GV37, family Accipitridae). The latter grouped together with two strains isolated from feral pigeons, which were separated from strains collected from pigeons of a pigeon loft [16].

**Fig. 2:**
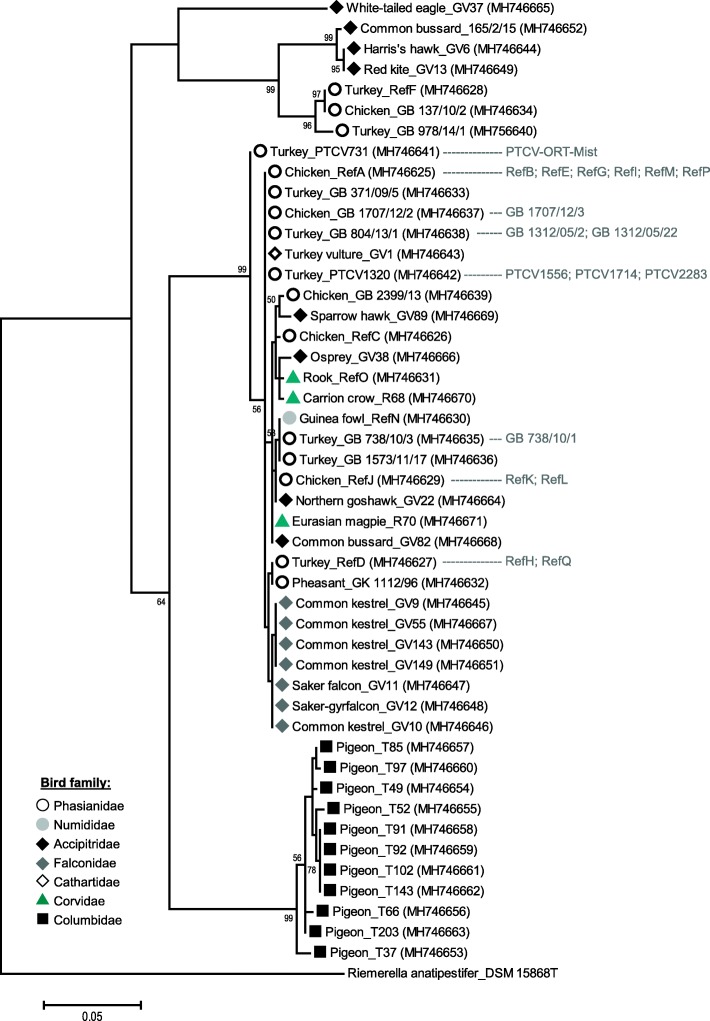
Phylogenetic tree based on partial *rpoB* gene sequences (538 bp) and constructed in MEGA6 [21] by using the Maximum Likelihood method based on the Jukes-Cantor model [22]. The tree was built with 47 out of 65 *O. rhinotracheale* sequences (remaining identical sequences are indicated in grey) and *Riemerella anatipestifer* DSM 15868^T^ (NC_017045) as outgroup. GenBank accession numbers are given in brackets. The percentage of replicate trees (> 50%) in which the associated taxa clustered together in the bootstrap analysis (100 replicates) is shown next to the branches. Initial tree(s) for the heuristic search were obtained by applying the Neighbor-Joining method to a matrix of pairwise distances estimated using the Maximum Composite Likelihood (MCL) approach. The tree is drawn to scale, with branch lengths measured in the number of substitutions per site

The 65 partial *rpoB* gene sequences were heterogeneous and ranged in their similarity values from 85.1 to 100%. The structure of the phylogenetic tree was in strong correlation with MLST results [16]. Three distinct clusters A (*n* = 6; poultry and birds of prey), B (*n* = 47; poultry, birds of prey and corvids) and C (*n* = 11; only pigeon) were identified (Fig. 2) that corresponded well to the MLST clusters [16]. Strain GV37 formed a distinct lineage and only shared 86.2 to 87.4% sequence similarity with strains of cluster A, 86.8 to 87.5% similarity with strains of cluster B and 85.1 to 85.5% similarity with strains of cluster C (Table 2). Within cluster A, three strains from turkeys and chicken (98.7 to 100% similarity) were clearly separated from three strains collected from birds of prey (99.4 to 100% similarity). Both groups shared only 94.2 to 94.8% sequence similarity. In contrast, *rpoB* gene sequence similarity values among strains from cluster B or cluster C ranged from 98.5 and 98.0% to 100%, respectively.

Sequence analysis of a specific *rpoB* gene fragment is widely used in addition to the 16S rRNA gene for more reliable bacterial identification and taxonomic classifications at genus and species level [17]. For members of the family Pasteurellaceae, *rpoB* similarity cut-offs of 85 and 95% were recommended for the description of new genera and species, respectively [23]. For the family Flavobacteriaceae, corresponding similarity cut-offs have not been defined so far [24]. Partial *rpoB* gene analyses, however, proved to be very useful to reveal clear phylogenetic relationships of *Riemerella* strains and related members of Flavobacteriaceae [19]. Likewise, *O. rhinotracheale* strains from this study show strong differences in similarity among their *rpoB* gene sequences. Strains of cluster A and cluster C as well as strain GV37 were clearly separated from cluster B, a genetically homogenous group that includes the majority of *O. rhinotracheale* strains and the *O. rhinotracheale* type strain DSM 15997^T^. Moreover, comparisons with *O. rhinotracheale* DSM 15997^T^ or respective with strains from cluster B revealed sequence similarities of ≤90.0% but above 85.0% (Table 2), which would only support bacterial identification at genus level.

## Conclusions

By using partial sequences from a single gene, the *rpoB* gene analysis is in good agreement with MLST results with a slight decrease in resolution to distinguish more similar strains. Eight *rpoB* gene sequences were received from swab samples (all non-galliform birds) [20] extending the avian host range and phylogenetic relationships investigated by MLST. On a bird family-level, the Columbidae specific cluster C and two different subclusters of three strains from Accipitridae species (namely 165–2/2015, GV8 and GV13) and seven strains from Falconidae species (namely GV9 to GV12, GV55, GV143 and GV149) were seen likewise to MLST [16]. At the same time, however, several *O. rhinotracheale* strains from different poultry species, birds of prey and corvid species have a close genetic relationship (similarity ≥98.5%) pointing towards the risk of interspecies transmission. Moreover, our results provide strong evidence that traditional phenotypic and genetic methods used for identification may not properly represent the heterogeneous group of bacteria classified as *O. rhinotracheale*. From housekeeping gene analyses, it is very likely that the genus *Ornithobacterium* includes additional species and partial *rpoB* gene sequencing can be recommended as fast, cost-effective and readily available method to identify strains and differentiate between *O. rhinotracheale* and *Ornithobacterium*-like bacteria.

## Abbreviations

MLST: Multilocus sequence typing
PCR: Polymerase chain reaction
ST: Sequence type
